# Physicochemical, Antioxidant and Mineral Composition of Cascara Beverage Prepared by Cold Brewing

**DOI:** 10.17113/ftb.63.01.25.8605

**Published:** 2025-03

**Authors:** Sali Muriqi, Libor Červenka, Lenka Česlová, Michal Kašpar, Soňa Řezková, Lenka Husáková, Jan Patočka, Petr Česla, Helena Velichová

**Affiliations:** 1 Department of Analytical Chemistry, Faculty of Chemical Technology, University of Pardubice, Studentská 573, Pardubice 53210, Czech Republic; 2 Department of Food Analysis and Chemistry, Faculty of Technology, Tomáš Bata University in Zlín, nám. T. G. Masaryka 5555, 460 01 Zlín, Czech Republic

**Keywords:** cascara, cold brewing, temperature effect, caffeine

## Abstract

**Research background:**

Cascara, the dried husk of coffee cherries, has attracted attention as a potential beverage due to its unique flavour profile and potential health benefits. Traditionally, cascara is prepared using hot brewing methods. However, recent interest in cold brewing methods has led to research on how temperature affects the functional properties of cascara beverages.

**Experimental approach:**

Colour (CIE L*a*b*), total dissolved solids and titratable acidity were determined in cascara beverages prepared at 5, 10, 15 and 20 °C. The concentration of phenols and flavonoids, as well as antioxidant properties were evaluated using spectrophotometric methods. Caffeine, chlorogenic acid and melanoidins were quantified by HPLC. The mineral composition was determined using inductively coupled plasma mass spectrometry (ICP-MS). The results were compared with a hot-brewed cascara beverage.

**Results and conclusions:**

Cold brewing resulted in significantly higher concentrations of total phenolic compounds, expressed as gallic acid equivalents (ranging from 309 to 354 mg/L), total flavonoids, expressed as quercetin equivalents (11.8–13.6 mg/L), and caffeine (123–136 mg/L) than the hot-brewed cascara beverage sample (p<0.05). Temperature had a noticeable effect on most variables, although the effect appeared to be random. In particular, concentrations of caffeine (p<0.01) and copper (p<0.001) were highest in beverages prepared at 20 °C and decreased with decreasing brewing temperature. Multivariate analysis showed that minerals (As, Co, Mn, Sn, Mg and Ca), hue and phenolic concentration contributed to the first principal component, which mainly differentiated the hot-brewed sample. Antioxidant-related variables, total titratable acidity and Se contributed most to the second principal component, which facilitated the separation of samples brewed at 5 °C.

**Novelty and scientific contribution:**

To our knowledge, this is the first study to suggest that temperature affects the functional properties of cascara beverage produced by the cold brewing method. Experimental evidence supports the existence of a direct proportionality between caffeine and copper concentrations and brewing temperature.

## INTRODUCTION

Coffee is one of the most widely consumed beverages with a worldwide production of more than 105 million tonnes per year (*1*). The production of coffee beans generates a lot of waste material. The presence of caffeine, polyphenols and tannins in coffee by-products leads to environmental problems in coffee-producing countries if they are disposed of improperly (*2*). In addition, its use as animal feed is limited due to similar factors. Coffee by-products are considered a sustainable source of beneficial bioactive compounds, including nutrients and other important substances.

Cascara, the main by-product of the coffee industry, has gained increased attention in the last decade due to its revalorisation (*3*). This unique beverage has tea-like properties and a distinct coffee aroma. Cascara is usually produced by drying the coffee pulp in the sun for 4 to 5 days, resulting in a blackish brown colour. In addition to its use as a beverage, cascara offers a potential source of phenolic compounds with antioxidant properties, which could have a beneficial effect on human health (*3*–*5*). It is also a rich source of minerals, including potassium, calcium, magnesium, sodium and iron (*6*).

Cascara is currently used for composting purposes in coffee-producing countries. This by-product has also been suggested for various applications, including the production of biofuel, enzymes, biosorbents and animal feed (*7*). Furthermore, there are proposals for its use in the food industry as an enrichment agent for functional foods such as gluten-free bread (*8*), yoghurt (*9*) and alcoholic beverages (*10*) due to its antioxidant properties, low fat, low sugar and high fibre content.

Cold brewing is a method of preparing beverages in which plant material is steeped in cold water for an extended period, usually between 8 and 24 h. Despite its popularity, there is still a lack of accurate information on the effects of cold brewing on the quality of cascara beverages. Recently, Abduh *et al.* (*11*) conducted the first and only comparative study evaluating the quality of cascara prepared using hot and cold brewing methods. To make a well-informed comparison, we will draw on established methods used in the preparation of coffee beverages (*12*–*18*).

When coffee is brewed in cold water, the resulting drink has different characteristics compared to its hot-brewed counterpart. Cold-brewed coffee tends to be less acidic, less bitter and significantly sweeter (*6*, *16*). It also contains less caffeine and brown compounds than its hot-brewed counterpart (*14*, *16*, *17*). However, the specific effects of cold brewing on cascara infusion are not unexplored. Several studies have been carried out to investigate the effect of low temperatures (*i.e.* <25 °C) on the final quality of coffee beverages (*12*–*17*). Research into the physical and chemical parameters of cascara beverages using this preparation method is intriguing. Coffee cherries, rich in simple sugars and free amino acids, undergo a combination of drying processes that can lead to the formation of various Maillard reaction products (*4*). In particular, dried coffee husks have recently been classified as safe by the European Commission in terms of acrylamide content (*19*). However, the effect of cold brewing on the content of furfural and 5-(hydroxymethyl)furfural (5-HMF), both substances of interest, in cascara infusion remains an open question. The European Food Safety Authority (EFSA) has set an acceptable daily intake (ADI) of 0.5 mg/kg body mass for furfural due to its potential liver toxicity. Additionally, EFSA provides a maximum permitted mass fraction of 10 mg/kg for 5-HMF in nonalcoholic beverages (*20*). Although these guidelines apply to other contexts, their relevance to cold-brewed cascara deserves further investigation (*21*, *22*).

While several studies have examined the cold brewing of coffee, there is a notable lack of research focusing specifically on cold preparation of cascara. To date, only one study has compared the methods of hot and cold brewing of cascara (*11*). This study aims to fill this gap by investigating the effects of different brewing temperatures on the quality of cascara beverages and set a new benchmark in the field of beverage science.

## MATERIALS AND METHODS

### Chemicals

The following substances were used to determine antioxidant activity: DPPH (1,1-diphenyl-2-picrylhydrazyl), ABTS (2,2´-azino-bis-(3-ethylbenzothiazoline-6-sulfonic acid), TPTZ (2,4,6-tri(2-pyridyl-*S*-triazin)) and Trolox ((±)-6-hydroxy-2,5,7,8-tetramethylchromane-2-carboxylic acid) (Sigma-Aldrich, Merck, St. Louis, MO, USA). The total phenolic content (TPC) was analysed using Folin-Ciocalteu´s phenol reagent (2 M) with gallic acid (purity ≥98 %) as a standard. Phenolic compounds (quercetin hydrate, chlorogenic acid), melanoidins (furfural and 5-(hydroxymethyl)furfural) and caffeine with a purity of at least 95 % were purchased from Sigma-Aldrich, Merck. Sodium hydroxide, sodium carbonate, hydrochloric acid, aluminium chloride hexahydrate, Fe(III) chloride tetrahydrate, sodium acetate, potassium persulfate and potassium chloride of American Chemical Society (ACS) grade were obtained from Lach-ner, s.r.o. (Neratovice, Czech Republic) . Methanol, acetonitrile (both gradient grade, Honeywell, Charlotte, NC, USA) and deionised water (Mili-Q system; Merck, Darmstadt, Germany) were used for the preparation of mobile phases in the HPLC analysis.

### The preparation of a cascara beverage

The dried coffee cherry husks were purchased from a local shop, vacuum sealed and kept at room temperature until further analysis. The sample originates from Hacienda Sonora (Costa Rica) and was harvested in 2022. For cold brewing, 9.0 g of coffee cherry husks were transferred to 300 mL of pre-tempered distilled water in a glass beaker covered with a watch glass and macerated at 5, 10, 15 and 20 °C (samples CB5, CB10, CB15 and CB20, respectively) for 24 h under constant stirring (200 rpm) using IKA^TM^ Plate RCT digital (IKA-Werke GmbH & Co. KG, Staufen, Germany). The solids were then removed using Whatman^TM^ filter paper (grade 1). For comparison purposes, a hot-brewed cascara beverage was prepared by mixing 9.0 g of cascara and 300 mL of boiled distilled water. Coffee cherry husks were macerated for 8 min and stirred at 90 s intervals with a glass rod (each stirring lasted 5 s). The temperature of the mixture decreased to (70±3) °C during maceration and the beverage was cooled in an ice bath. Each sample was prepared in triplicate and each of them was analysed at least twice. After the removal of solids by filtration, the liquid samples were used to determine the colour, total titratable acidity and total dissolved solids. The samples were kept in small aliquots at -25 °C for further analysis.

### Colour, total titratable acidity and total dissolved solids of cascara beverages

The colour was measured in reflectance mode on a 50-mm path quartz cuvette using an UltraScan VIS spectrophotometer (Hunter Associates Laboratory, Reston, VA, USA). The CIELAB colour space was applied to describe L* [dark (0) to light (100)], a* [red (+) to green (-)], and b* [yellow (+) to blue (-)]. The hue angle (*h°*) and the chroma (C*_ab_) of the colour were also determined. Total titratable acidity (TTA) was determined using a titration method. An aliquot of 50.0 mL of cascara brew was titrated with a 0.1 M NaOH solution to a pH=8.1 using the pH glass electrode HC 103 (Theta 90, Prague, Czech Republic) (*23*). The results were expressed in millilitres of NaOH per litre. Total dissolved solids (TDS) of hot- and cold-brewed cascara beverages were measured according to the protocol of Moreno *et al.* (*24*). The °Brix of each beverage was measured using an AR3/AR4 refractometer (Mettler-Toledo, Greifensee, Switzerland) followed by conversion to TDS using the following equation:


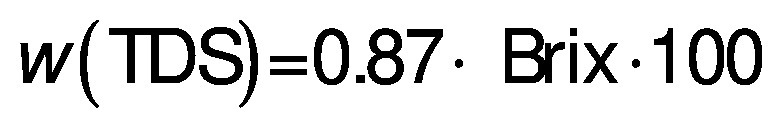
 /1/

### Determination of total phenolic and total flavonoid concentrations

The total phenolic concentration (TPC) was determined using the Folin-Ciocalteu´s reagent according to a method from our previous study (*25*). The absorbance was monitored after 30 min at 765 nm (UV-2600; Shimadzu, Kyoto, Japan) and the results were expressed in mg of gallic acid equivalents per litre of beverage. The ability of aluminium chloride to form complexes with flavonoids in an acidic environment was used to determine the total flavonoid concentration (TFC) using a protocol described in our previous work (*25*). The formation of Al^3+^-flavonoid complexes was determined at 425 nm and the results were expressed as concentration of quercetin equivalent in mg/L.

### Antioxidant properties of cascara beverages

The radical scavenging assays using stable DPPH and ABTS radicals were adopted from our previous study (*25*). The absorbance of the samples was measured at 734 and 517 nm for the ABTS and DPPH assays, respectively. The results were expressed as Trolox equivalent antioxidant capacity (TEAC in mg/L). The Fe(III) reducing antioxidant power (FRAP) was determined after the reaction of the sample with TPTZ solution and FeCl_3_ in an acidic environment (*26*). FRAP values were expressed concentration of Trolox in mg/L.

### Determination of the total melanoidin concentration

The melanoidin concentration in hot- and cold-brewed cascara beverages was measured spectrophotometrically at 420 nm in a quartz cuvette with a path length of 10 mm. Melanoidins were quantified using the calibration curve for caramel (E-150d) in the range of 0.19–6.66 g/L and the results were expressed as caramel concentration in mg/L (*4*).

### HPLC analysis of target compounds

Caffeine and chlorogenic acid isomers (chlorogenic, neochlorogenic and cryptochlorogenic) were analysed using a liquid chromatography system consisting of two high-pressure pumps LC-20ADXR, a degassing unit DGU-20A3R, an autosampler SIL-20ACXR, a photodiode array detector SPD-M30A (all Shimadzu, Kyoto, Japan) and a column thermostat LCO 102 (Ecom, Prague, Czech Republic). Separation was carried out on the YMC-Triart C18 chromatographic column (150 mm×3 mm, *d*(particle)=3 μm) with the mobile phase consisting of deionised water acidified with 0.3 % (by volume) formic acid (solvent A) and methanol (solvent B). The optimal gradient program was as follows: 0 min at 10 % B, 6 min at 33 % B, 9 min at 33 % B, 13 min at 55 % B, 18 min at 90 % B and 20 min at 10 % B. The mobile phase flow rate, injection volume and column temperature were set to 0.4 mL/min, 2.0 μL and 30 °C, respectively. Detection wavelengths of 273 and 325 nm were used for the monitoring of caffeine and chlorogenic acid isomers, respectively (*27*).

The Agilent 1200 Series liquid chromatography system was used for the determination of furfural and 5-HMF in cascara beverages (Agilent Technologies, Santa Clara, CA, USA), adopting a method of Czerwonka *et al.* (*22*) with some modifications. Separation was carried out on a Luna^®^ Omega Polar column (150 mm×3 mm, *d*(particle)=3 μm) with the mobile phase consisting of deionised water (solvent A) and acetonitrile (solvent B), both acidified with 0.1 % (by volume) acetic acid. The optimal gradient program was as follows: 0 min at 0 % B, 8 min at 8 % B, 8.1 min at 100 % B, 12 min at 100 % B, 12.1 at 0 % B and 17 min at 0 % B. The mobile phase flow rate, injection volume and column temperature were set to 0.5 mL/min, 15.0 μL and 40 °C, respectively. Both compounds were detected at a wavelength of 280 nm.

Quantitative analysis of all chlorogenic acid isomers was performed using a chlorogenic acid standard (5-*O*-caffeoylquinic acid) and is presented as their sum. Caffeine, furfural and 5-HMF were quantified using the corresponding standards. Seven calibration solutions of chlorogenic acid and caffeine were prepared by sequential dilution of their methanolic stock solutions (1.0 g/L) with 20 % (by volume) aqueous methanol to concentrations ranging from 5-100 mg/L for caffeine and 0.5–10 mg/L for chlorogenic acid. Six calibration solutions (0.1–10.0 mg/L) of furfural and 5-HMF were prepared by diluting their stock solutions in 50 % (by volume) aqueous methanol. All samples were filtered through a 0.45-μm PTFE syringe filter before injection.

### Mineral composition of cascara beverages

The multi-element quantification of major (K, Ca, P, Mg and Na) and trace (Fe, Mn, Cu, Zn, Se, Ni, Co, Cr, Al, As, Cd and Pb) elements was performed using the Agilent 7900 ICP-MS (Agilent Technologies, Inc.). The instrument was configured with standard nickel cones, a glass concentric nebulizer (MicroMist, 400 µL/min), a Peltier-cooled quartz spray chamber (2 °C) and a quartz torch with a 2.5-mm internal diameter. A 10-roller peristaltic pump with low pulsation and three separate channels ensured accurate delivery of both samples and the internal standard. To mitigate polyatomic interferences, the instrument included an octopole-based collision cell using kinetic energy discrimination (KED) in either standard helium or high-energy helium mode. The collision cell parameters were set manually for both modes and the MassHunter software of the instrument tuned them automatically at start-up to enhance the sensitivity for elements with different mass-to-charge ratios (low, medium and high). The tuning parameters for plasma and ion lens were kept constant for all collision cell modes (for detailed information, see Varrà *et al.* (*28*)).

The determination of analyte concentrations involved the construction of calibration curves covering multiple elements. These curves were established by analysing calibration solutions containing standards at five different concentrations. The concentration ranges for the different elements were as follows: 0 to 100 μg/L for Fe, Mn, Cu, Zn, Se, Ni, Co, Cr, Al, As, Cd and Pb, and 0 to 10 mg/L for K, Ca, P, Mg and Na. Linear calibrations with a coefficient of determination of more than 0.999 were successfully achieved for all analysed elements. To account for possible instrumental drift and matrix effects, a 200 µg/L Rh internal standard was simultaneously introduced and mixed with the samples. The applied method was comprehensively validated using certified reference standards. The results of this rigorous validation process are detailed in the work by Varrà *et al*. (*28*).

### Statistical analysis

Each brewing was performed in triplicate and the results were expressed as the mean value with standard deviation. The homogeneity of variance and the normal distribution of the data were tested using the Box's M test and the Shapiro-Wilk's test, respectively. Analysis of variance (ANOVA) was used to study the effect of brewing temperature on the cold-brewed samples. A *post-hoc* Duncan’s test was used for multiple pairwise comparison among means. Pearson’s correlation (r) analysis was used to calculate the pairwise correlation coefficient matrix with the corresponding significance test p-values between pairs of variables. In all cases, a p<0.05 was chosen as the statistical significance threshold.

Hidden patterns in the data were uncovered by applying multivariate analysis. The most informative variables were selected in advance to simplify the final multivariate models. Neighborhood component analysis (NCA) was used for this purpose (*28*). Ddifferences between the analysed groups of cascara samples were assessed using principal component analysis (PCA). Hierarchical cluster analysis (HCA) was additionally applied to the same input data used for PCA, namely a matrix consisting of the normalised yields obtained from each measurement. For HCA, an agglomerative hierarchical algorithm was used that progressively combines pairs by measuring Euclidean distances among the clusters. The average linkage method was selected as it provided the highest cophenetic correlation (0.98).

All statistical analyses were carried out using the software packages MATLAB® R2022b (*29*) and Statistica v. 14.0 (*30*).

## RESULTS AND DISCUSSION

### The effect of brewing temperature on dissolved solids, titratable acidity and colour of cascara beverage

The results are summarised in Table 1. The values of total dissolved solids (TDS) were highest for the CB20 sample ((0.4±0.1) %) and decreased with decreasing temperature, exactly as reported for the coffee beverage (*13*). They found that TDS after brewing were higher at 30 °C and lower at 4 °C, depending on other variables such as coffee-water ratio, mesh size and extraction time. In our study, the TDS values were not significantly different in cascara samples brewed at 15 to 5 °C and were similar to the hot-brewed sample (0.19±0.05) %. Hot water is known to be more effective in the dissolution of chemical substances and/or the breakdown of tea polymers, which increases the turbidity of solutions and thus the total soluble solid content (*31*). Despite this fact, Zhao *et al*. (*32*) found that hot (85 °C for 25 min) and cold brews (4 °C for 9 h) of *Apocynum venetum* tea had a similar solid content. Furthermore, their research showed that tea brewed at room temperature (25 °C for 3 h) had significantly lower total soluble solids.

**Table 1:** Total dissolved solids (TDS), total titratable acidity (TTA) and colour of cascara beverages

Parameter	Hot-brewed	Cold-brewed at *t*/°C				p
		20	15	10	5	
*w*(TDS)/%	(0.19±0.05)^b^	(0.4±0.1)^a^	(0.27±0.05)^b^	(0.20±0.03)^b^	(0.26±0.01)^b^	*
*φ*(TTA)/(mL/L)	(31.9±1.8)^bc^	(34.1±1.0)^a^	(32.8±0.2)^b^	(33.±0.4)^ab^	(30.7±0.4)^c^	**
L*	(63.1±1.8)^b^	(45.3±4.7)^c^	(45.9±4.4)^c^	(37.7±1.6)^d^	(80.9±3.4)^a^	**
a*	(26.6±1.9)^bc^	(28.6±0.7)^b^	(29.3±1.2)^b^	(26.0±1.0)^bc^	(53.4±1.8)^a^	**
b*	(82.2±1.3)^b^	(70.5±5.5)^c^	(71.7±5.2)^c^	(60.2±2.1)^d^	(109.9±0.2)^a^	*
*h*°	(72.1±0.9)^a^	(67.9±1.2)^b^	(67.8±1.2)^b^	(66.7±0.8)^b^	(64.1±0.8)^c^	***
C*_ab_	(86.4±1.8)^b^	(76.1±5.4)^c^	(77.5±5.1)^c^	(65.6±2.1)^d^	(122.2±0.9)^a^	***

Mean value±standard deviation (*N*=3). Different lower-case letters in superscript in the same row indicate statistical differences using Duncan´s multiply pairwise test (p<0.05). p=probability factor indicating the significance of cold brewing temperature on the variables: *p<0.05, **p<0.01 and ***p<0.001

The TTA values decreased from (34.1±1.0) to (30.7±0.4) mL/L with the decrease in brewing temperature (p<0.001). The hot-brewed sample showed similar acidity to those prepared at 5–15 °C in our study. Generally, hot water extraction is more efficient in dissolving and transferring some acidic species, as shown in the case of Colombian or arabica coffee infusions (*14*, *17*).

In our study, the cold-brewing method was supported by constant stirring, which was probably more effective for the release of substances of an acidic nature. The colour of the hot-brewed cascara beverage differed in the parameters L* and b* from those prepared in cold water (Table 1). The final beverages prepared at 20, 15 and 10 °C were darker and less yellow. The sample prepared at 5 °C had a high red colour (a*=53.4±1.8), high yellow (b*=109.9±0.2), and high degree of lightness (L*=80.9±3.4). Recently, the colour of the cascara beverage was attributed to the presence of melanoidins that develop during the drying of cherry pulp (*4*). This was also confirmed in our experiment, where the melanoidin content of the hot-brewed samples and the samples brewed at 20, 15 and 10 °C was negatively associated with the colour values L* and b*. In other words, the more melanoidins, the darker (r=-0.745; p<0.01) and less yellow (r= -0.621; p<0.05) the beverage.

### Antioxidant properties, phenolic and caffeine concentration in cascara beverage

**Fig. 1:**
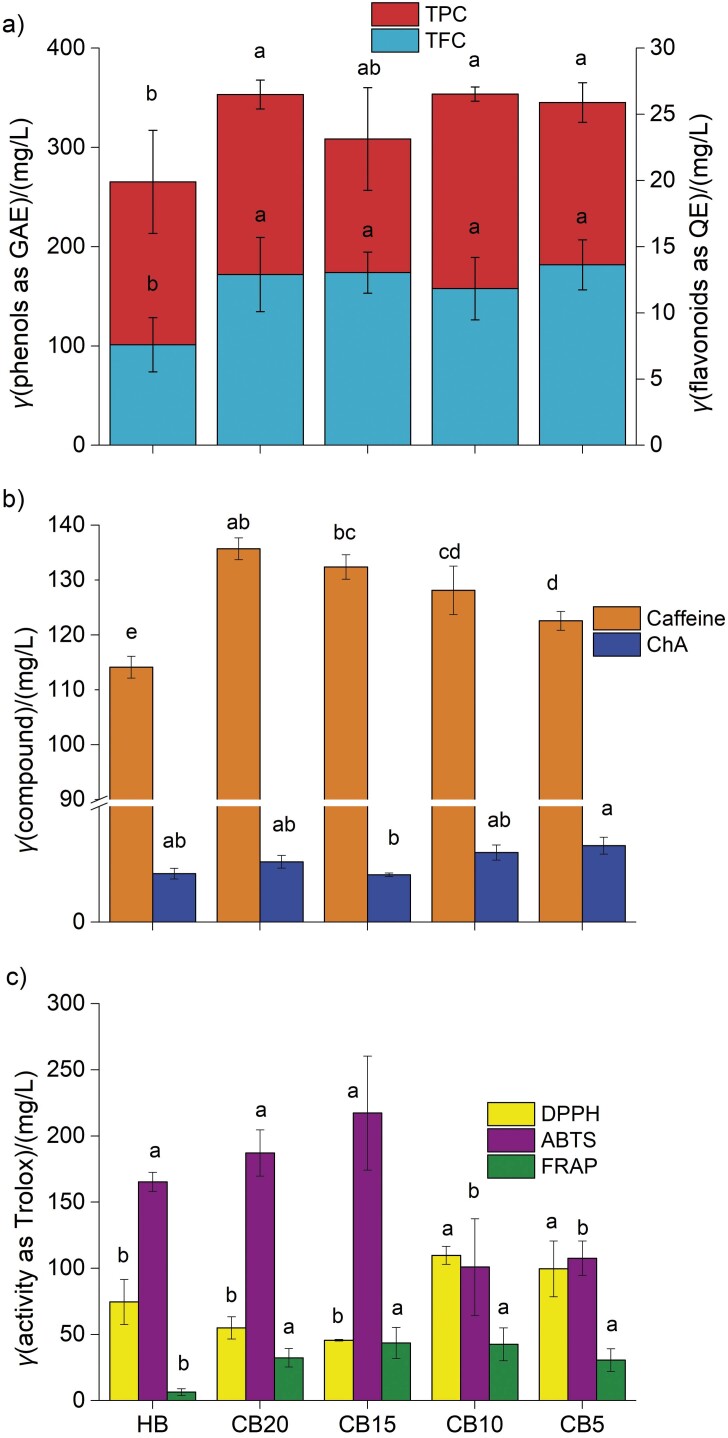
Determination of phenols and antioxidants in cascara beverages prepared by hot (HB) and cold brewing at 20, 15, 10 and 5 °C (CB20, CB15, CB10 and CB5): a) total phenolic (TPC expressed as gallic acid equivalents (GAE)) and total flavonoid concentrations (TFC expressed as quercetin (QE)), b) chlorogenic acid (ChA) and caffeine concentrations, c) antioxidant activity. Mean value±standard deviation (*N*=3). Different lower-case letters above the bars for each variable indicate a significant difference at p<0.05

In general, both total phenolic and total flavonoid concentrations were higher (p<0.05) in the cold-brewed cascara beverage samples than in the hot-brewed samples (Fig. 1a). While the TPC of the cold-brewed cascara, expressed as gallic acid equivalents (GAE), was 309–354 mg/L, the hot-brewed sample yielded (265±52) mg/L. Heeger *et al*. (*2*) determined a similar total phenolic content (283 mg/L) in a cascara beverage by extracting 1.0 g of coffee cherry pulp in 24 mL of hot water at 85 °C for 15 min. The data comparing the TPC of hot-brewed and cold-brewed cascara beverages is insufficient. Abduh *et al*. (*11*) suggested in their preliminary experiment that TPC was higher in hot-brewed than in cold-brewed cascara although they did not provide specific numerical values. Numerous studies have investigated the effects of hot and cold brewing methods on different beverages, but their results are inconsistent and vary widely (*12*–*16*). For example, the total phenolic content was higher in hot-brewed (92 °C for 3 min) than in cold-brewed (5 °C, 16 h) coffee (*12*). This is usually explained by a higher solubility of some phenols, which leads to an increase in their concentration in the final beverage. On the other hand, French press and cold brewing (room temperature for 12 h or 4 °C for 24 h) produced a coffee beverage with a similar TPC (*13*). It should be noted that the extraction efficiency of phenolic substances from ground coffee beans is a complex process that involves not only the temperature of the water (or the brewing method), but also the brewing time, grinding size, the origin of the beans and the degree of roasting (*13*, *15*). In a recent study by Muzykiewicz-Szymańska *et al*. (*15*), a higher content of polyphenols was found in unroasted coffee bean infusion prepared with cold water (4 °C for 24 h) than that prepared using hot brewing methods (combinations of 86 and 95 °C for 4 or 10 min). Non-fermented and partially fermented teas had higher TPC when brewed at low temperature (4–6 °C for 16 h) than when prepared with boiled water (*33*). However, in their study, the opposite trend was observed in fermented teas. They proposed that non-fermented tea leaves contain low-molecular-mass water-soluble phenols that are easily extractable in cold water. In contrast, fermentation leads to the formation of polymeric products of higher molecular mass that are more soluble in hot water. Similarly, the total flavonoid concentration, expressed as quercetin equivalents, of all samples of the cold-brewed cascara beverages was higher (11.8–13.6 mg/L) than of the hot-brewed cascara beverage ((7.6±2.1) mg/L). There are two possible explanations for this phenomenon. The coffee husk is usually dried in the sun and, as described for the cornelian cherries, the contribution of free phenols was more dominant in the sun-dried samples than in the fresh samples (*34*). It should also be mentioned that the TPC values may be overestimated due to the reaction of melanoidins with Folin-Ciocalteu´s reagent (*27*). As described below, the cold-brewed samples had a higher concentration of total melanoidins than the hot-brewed cascara sample. The effect of temperature on TPC and TFC during cold brewing has not been proven (p>0.05). Among the phenolic compounds, protocatechuic acid, pyrocatechol and chlorogenic acid were the most abundant in five cascara samples, as observed by Pua *et al*. (*5*). The chlorogenic acid concentration ranged from 31.6 to 256 mg/L, which was considerably higher than that found for both hot- and cold-brewed cascara beverages in our study, *i.e*. 8.4–13.2 mg/L (Fig. 1b).

This discrepancy can be attributed to the low solid-liquid ratio. We prepared cascara at a ratio of 3:100, while Pua *et al*. (*5*) used more dried coffee husks (1:10). Additionally, both hot- and cold-brewed cascara beverages had similar concentrations of chlorogenic acid (p>0.05). Regarding the caffeine concentration, the cold-brewed cascara had a significantly higher amount (123–136 mg/L) than the hot-brewed sample (p<0.01). The effect of water temperature on the chlorogenic acid and caffeine concentration in cascara beverage has not yet been described. However, there are some studies that compare the concentrations of these substances in cold and hot coffee drinks (*6*, *14*, *27*). The high solubility of neochlorogenic acid in water was probably responsible for facilitating its extraction at low (21–25 °C, 24 h) and high (98 °C for 6 min) temperatures, as demonstrated in Arabica coffee brews (*35*). They also found that the caffeine concentration was higher with long maceration at low temperature than with fast and hot extraction, which was attributed to the limitation of diffusion of caffeine through the particles with a larger radius. This was similar to our study. Furthermore, the decrease in temperature from 20 to 5 °C resulted in a decrease in the caffeine concentration from 136 to 123 mg/L (p<0.01) in cascara beverages. The extraction efficiency of chlorogenic acid and caffeine from coffee beans depends on a number of factors such as water temperature, extraction time, particle size, origin of the coffee and degree of roasting. For example, a coarse grinding and a higher extraction temperature (15 °C) favoured the caffeine content of Arabica coffee brew, while the extraction of fine particles at 5 °C was more effective for Robusta coffee (*6*). A higher caffeine concentration in a coffee drink prepared at 25 °C than at 15 °C and 5 °C was also found in the study by Maksimowski *et al*. (*18*), but only for a coffee roasted at 220 °C. This shows that the extraction of caffeine and phenols from the plant matrix is a complex process. Further studies are needed to reveal the reasons for the decrease in caffeine concentration with decreasing temperature.

The antioxidant properties, expressed as Trolox equivalents (TE), of the hot-brewed cascara sample were 165 and 75 mg/L in terms of the ABTS and DPPH assays, respectively (Fig. 1c). While the ABTS method resulted in similar TEAC values for the samples prepared at 20 and 15 °C, further decrease in the brewing temperature caused a significant decrease in antioxidant activity (p<0.01). On the contrary, the DPPH radical scavenging activity of cascara beverage samples brewed at 20 and 15 °C was lower than of the hot-brewed sample, although the differences were not significant at p>0.05. Lowering the soaking temperature to 10 and 5 °C resulted in the cascara beverage with significantly higher TEAC_DPPH_ values (p<0.01). Therefore, a negative correlation was observed between TEAC_DPPH_ and TEAC_ABTS_ (r=-0.685, p<0.01) in this study. It is known that the ABTS assay is more sensitive to hydrophilic and lipophilic compounds, while the DPPH radical scavenging assay is suitable for the analysis of hydrophobic compounds (*36*). They found a negative association between the ABTS and DPPH radial scavenging ability in the Umbelliferae plant. In a study by Yi *et al.* (*37*), some phenols (chicoric, chlorogenic and 3,5-dicaffeoylquinic acids) determined in *Lactuca indica* L. extracts showed a negative correlation coefficient for ABTS. Although the correlation coefficients were not significant at p>0.05, it was weakly positive between TPC and DPPH (r=0.254) and weakly negative between TPC and ABTS (r=-0.445). FRAP values (as TE) were higher for samples prepared at 15 and 10 °C (42.5–43.6 mg/L) than at 20 and 5 °C ((32.0±7.0) and (31.0±9.0) mg/L). However, the mean values were not different due to the high standard deviations (p>0.05). Hot brewing resulted in a cascara beverage with significantly lower FRAP value (p<0.01).

### Melanoidin concentration in hot- and cold-brewed cascara beverages

Although the total melanoidin content expressed as caramel was (3.4±0.3) g/L for the hot-brewed cascara beverage, cold brewing resulted in significantly higher concentrations ranging from (4.9±0.4) to (5.1±0.2) g/L (p<0.05) (Table S1). Iriondo-DeHond *et al*. (*4*) first reported the presence of melanoidins, expressed as caramel, in the coffee cascara at a concentration of 1480 mg/L. However, in their study, three times less instant coffee husk powder was used to prepare the beverage. In our study, temperatures below 20 °C did not influence the melanoidin concentration in cascara. 5-HMF and furfural accounted for only a small part of the total melanoidin concentration. We observed only 0.16–0.20 and 0.91–0.97 mg/L of furfural and 5-HMF, respectively. This is in agreement with the study conducted in coffee pulp distillate (*21*), where the authors showed that the furfural and 5-HMF concentration of 0.17 and 0.11 mg/L, respectively, was below the detection limit. In general, hot brewing resulted in more brown compounds or higher 5-HMF concentration in coffee beverages than cold brewing (*16*, *17*). Our study showed that neither compound showed any significant trend with temperature, although the pairwise comparison test indicated some significant differences. The total melanoidin concentration was only positively associated with 5-HMF (*r*=0.658, p<0.01) but not with furfural. Both hot- and cold-brewed cascara beverages can be considered safe due to the low 5-HMF content (*20*).

### Mineral concentration of coffee cascara beverages

The concentration of the elements in the hot- and cold-brewed cascara is listed in Table 2. Potassium, phosphorus, magnesium, calcium and sodium were found in a range of 507–594, 29.2–36.9, 6.4–12.0, 7.3–20.1 and 0.72–1.3 mg/L, respectively. Among the trace elements, manganese (102.0–140.0 μg/L), iron (112.0–143.0 μg/L) and copper (64.1–111.0 μg/L) occurred at the highest concentrations in all samples. Zinc was found only in the hot-brewed cascara beverages at a concentration of (42.0±2.0) μg/L, while it was below the detection limit in the cold-brewed cascara beverages. Cold brewing for 24 h improved the extraction of all minerals, resulting in their significantly higher concentration than in the hot-brewed sample. Although the effect of temperature on most minerals during cold brewing was found to be significant, no trend in mineral contcentration was evident with the brewing temperature. The exception is the decrease in copper concentration from (111.0±3.0) to (79.7±0.8) μg/L when the temperature decreased, and the lowest K concentration ((552±5) mg/L) at 5 °C. It has been previously shown that the coffee making technique has an influence on the mineral concentration (*38*). For example, the highest Mn concentration was found in Aeropress coffee and the lowest concentration in the coffee from the French press. Cu was below the detection limit in Aeropress and drip brews, but espresso coffee contained 85 μg/L of copper, which is similar to our study. Regarding the daily intake recommendation, cascara brews can serve as a source of some minerals. For potassium, an adequate intake (ADI) of 3.4 and 2.6 g per day was established for adult men and women, respectively (*39*). When consuming two cups a day (approx. 360 mL), the daily limit is reached by 6.3 % (male) and 8.2 % (female). Considering the reference daily intake (RDA) for Cu, which is 900 μg/day, two cups of cascara beverage prepared at 20 °C can provide up to 4.4 % of the daily requirement for this mineral (*40*). Manganese is involved in the formation of bones and is a co-factor in many enzymes. It is recommended to ensure ADI of 2.3 and 1.8 mg for men and women, respectively (*38*), only 2.2 and 2.8 % of which would be met by drinking two cups of cold-brewed cascara beverage (prepared at 10 °C). The same amount of cascara beverage would provide approx. 1 % or less of the RDA or ADI for other minerals determined in this study. Instant cascara powder is recognised as a source of potassium and magnesium because it provides at least 15 % of the daily recommendation (Regulation (EC) No 1925/2006) (*4*, *41*). Cascara beverages prepared at low temperature (especially at 10 °C) can be considered as a source of potassium only if a larger volume is consumed per day (860 mL for men and 660 mL for women). The aluminium and tin content was higher in the cold-brewed cascara samples, but their content peaked at different temperatures (15 °C for Al and 20 °C for Sn). On the other hand, cold brewing reduced arsenic or lead concentrations.

**Table 2:** The concentration of selected elements in hot and cold brewed cascara beverage

		Hot-brewed	Cold-brewed at *t*/°C				
Element			20	15	10	5	p
		*γ*(element)/(mg/L)					
K	(507.0±1.3)^d^		(577±11)^b^	(573±5)^b^	(594±8)^a^	(552±5)^c^	**
P	(29.2±0.6)^c^		(35.4±0.2)^a^	(35.7±0.8)^a^	(36.9±1.5)^a^	(33.3±0.8)^b^	**
Mg	(6.40±0.02)^c^		(10.6±1.1)^b^	(10.2±0.2)^b^	(12.0±0.6)^a^	(11.7±0.4)^ab^	*
Ca	(7.3±0.2)^d^		(16.6±1.9)^b^	(12.9±0.9)^c^	(19.9±1.7)^a^	(20.0±1.0)^a^	***
Na	(0.80±0.01)^c^		(1.1±0.1)^a^	(0.72±0.02)^c^	(1.07±0.08)^a^	(1.3±0.2)^a^	**
	*γ*(element)/(μg/L)						
Fe	(112.0±0.8)^b^		(133.2±4.0)^a^	(112.2±6.4)^b^	(130±29)^a^	(143.1±3.0)^a^	NS
Mn	(102.3±0.4)^c^		(134.8±3.1)^ab^	(121±10)^b^	(139.6±3.9)^a^	(127.1±2.0)^b^	*
Cu	(64.1±1.8)^d^		(111.0±3.2)^a^	(97.9±7.1)^b^	(90.3±9.1)^bc^	(79.7±0.8)^c^	**
Co	(0.586±0.001)^a^		(0.424±0.001)^e^	(0.474±0.001)^c^	(0.537±0.001)^b^	(0.436±0.001)^d^	***
As	(0.67±0.03)^a^		(0.19±0.01)^c^	(0.23±0.04)^bc^	(0.18±0.03)^c^	(0.25±0.02)^b^	NS
Se	(0.32±0.01)^b^		(0.41±0.03)^a^	(0.208±0.008)^d^	(0.35±0.01)^b^	(0.26±0.03)^c^	***
Cr	(0.112±0.003)^d^		(0.18±0.01)^b^	(0.15±0.01)^c^	(0.150±0.005)^c^	(0.36±0.01)^a^	***
Sn	(0.23±0.01)^e^		(1.797±0.001)^a^	(0.972±0.001)^c^	(0.766±0.001)^d^	(1.008±0.001)^b^	***
Al	(0.11±0.01)^b^		(0.18±0.02)^a^	(0.20±0.01)^a^	(0.18±0.02)^a^	(0.191±0.002)^a^	NS
Cd	(0.24±0.02)^b^		(1.20±0.02)^a^	(0.10±0.03)^c^	<LOQ	(0.25±0.03)^b^	ND
Pb	(3.7±0.2)^a^		(0.3±0.3)^b^	<LOQ	<OQ	<LOQ	ND
Ni	2.54±0.06		<LOQ	<LOQ	<LOQ	<LOQ	ND
Zn	42.0±2.0		<LOQ	<LOQ	<LOQ	<LOQ	<LOQ

Mean value±standard deviation (*N*=3). Different lower-case letters in superscript in a same row indicate statistical differences using Duncan´s multiple pairwise test (p<0.05). LOQ=limit of quantification. p=probability factor indicating the significance of cold brewing temperature on the variables: *p<0.05, **p<0.01, ***p<0.001, NS=not significant, ND=not determined

### Multivariate analysis of hot- and cold-brewed cascara samples

**Fig. 2:**
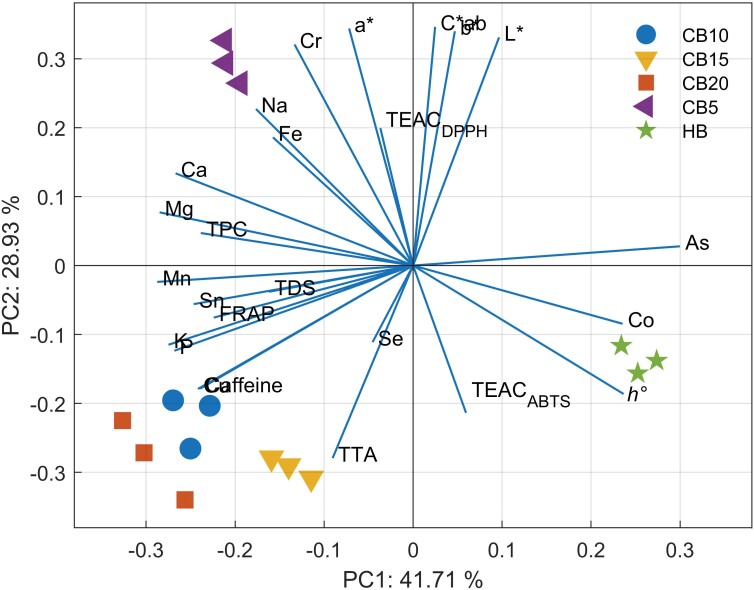
Biplot illustrating the principal component analysis of analyte profile in the cascara beverage samples prepared by hot (HB) and cold brewing at 5, 10, 15 and 20 °C (CB5, CB10, CB15 and CB20). TDS=total dissolved solids, TTA=total titratable acidity

**Fig. S1:**
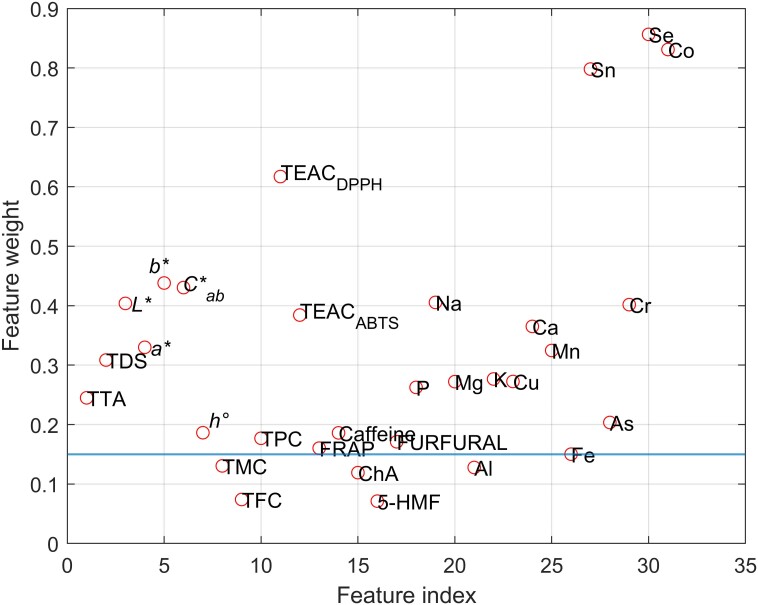
Plot of the feature weights obtained from the neighbourhood component analysis (NCA) feature selection algorithm for PCA classification: a relative threshold of the weights of the irrelevant features was set <0.15. ChA=chlorogenic acid, TDS=total dissolved solids, TFC=total flavonoid concentration, TPC=total phenolic concentration, TMC=total melanoidin content, TTA=total titratable acidity, 5-HMF=5-(hydroxymethyl)furfural

**Fig. S2:**
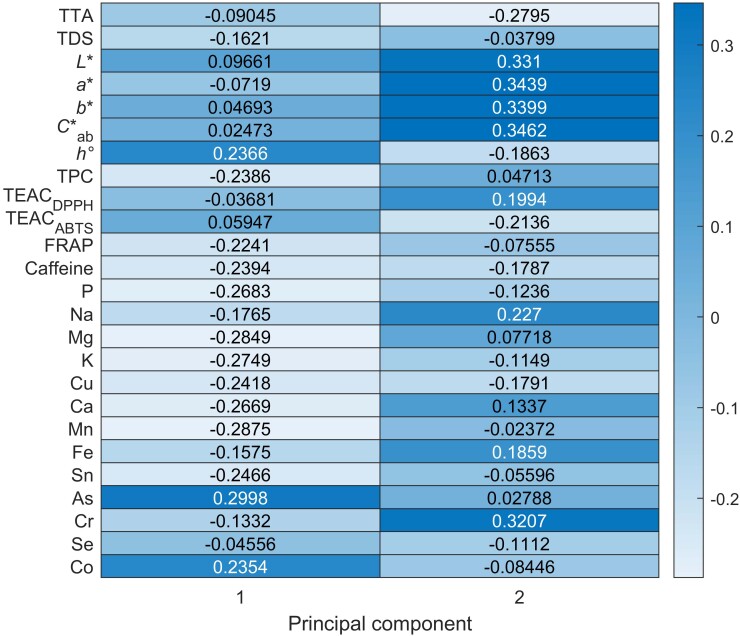
Heat map of the analyte loadings on the two components. TTA=total titratable acidity, TDS=total dissolved solids, TPC=total phenolic concentration

Before building the final model, the most informative variables were selected using neighbourhood component analysis (NCA), which allowed us to simplify the multivariate models while retaining key information. The NCA results (see Fig. S1) showed that specific analytes, with the exception of TFC, melanoidins (TMC, furfural and 5-HMF) and Al contribute significantly to the dataset. These analytes were included into the development of the PCA model designed to distinguish the hot-brewed sample from the cold-brewed samples and the cold-brewed samples at different temperatures. The first two principal components (PCs) represent 71 % of the total variability in the analyte profiles (Fig. 2). Importantly, the variables As, Co, Mn, Sn, Mg, Ca, hue and TPC showed strong contributions to the first principal component (PC1), which was primarily responsible for distinguishing hot-brewed samples. On the other hand, the characteristics of L*, a*, *C*_ab_, TEAC_ABTS_, TEAC_DPPH_, TAA and Se were the main contributors to PC2, leading to the separation of CB5 samples. The significance of these results lies in the fact that specific elemental and phenolic components play a crucial role in defining the unique analyte profiles of hot- and cold-brewed samples. For example, the contributions of heavy metals (As, Co, Mn and Sn) to PC1 suggest that these elements may be important for distinguishing hot-brewed sample, possibly due to differences in soil composition or cultivation conditions between hot- and cold-brewed sources. Meanwhile, the influence of antioxidant variables (TEAC and TAA) in PC2 on CB5 samples highlights the potential effects of different temperature treatments on the antioxidant properties of cold-brewed samples, which is consistent with previous studies showing that temperature can significantly affect the antioxidant profile of coffee beans (*12*-*15*). More detailed information can be found in Fig. S2, where the contributions of each variable to the principal components are shown as a heat map. This figure clearly shows the relative influence of each analyte on the different principal components, allowing a more nuanced understanding of their role in distinguishing between hot- and cold-brewed samples.

**Fig. S3:**
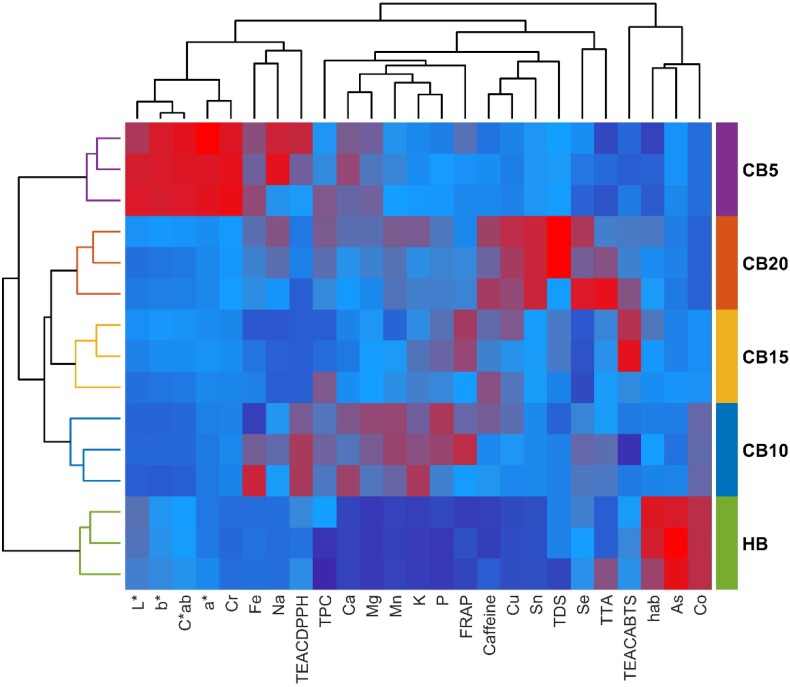
Dendrogram of hierarchical clustering analysis (HCA) and heat map of analyte concentrations of cascara samples/species at brewed at different temperatures. HB=hot-brewed, CB5, CB10, CB15 and CB20=cold-brewed at 5, 10, 15 and 20 °C, respectively. TPC=total phenolic concentration, TDS=total dissolved solids, TTA=total titratable acidity

Moreover, hierarchical cluster analysis (HCA) resulted in a similar grouping of samples as PCA, with five distinct clusters emerging naturally from the data (Fig. S3). This emphasises the robustness of the PCA model and suggests that the distinguishing features identified by PCA are consistent among different multivariate approaches. The agreement between PCA and HCA further emphasises the reliability of our future selection process and the stability of the identified variables in distinguishing different sample groups. The HCA dendrogram (Fig. S3) and the accompanying heat map visually represent the analyte concentrations in hot- and cold-brewed samples at different temperatures. This clustergram plays an important role in confirming the natural separation between the sample groups and highlights the different analyte profiles under different brewing conditions. The heat map provides a clear visualisation of the relative concentrations of key analytes and highlights patterns that may not be immediately evident from PCA alone. The dendrogram further confirms the results from PCA by showing the hierarchical relationships between samples with clear divisions based on temperature and preparation method. This consistency among different multivariate techniques highlights the robustness of the overall data analysis and strengthens the conclusions about the influence of temperature on the analyte profiles of hot- and cold-brewed samples.

To understand the broader significance of these findings, future studies could further investigate how these key variables, particularly metal elements and antioxidant compounds, correlate with the sensory properties or health benefits of hot- and cold-brewed samples. Additionally, extending the analysis to larger data sets or different geographical regions could provide further validation of these distinguishing factors and enhance the generalisability of our model.

## CONCLUSIONS

Cascara beverages prepared at low temperatures had a different colour and a higher concentration of total phenols and flavonoids than the hot-brewed sample. Higher antioxidant activity was demonstrated in all cold-brewed samples only by FRAP. Cold brewing also resulted in a beverage with a higher concentration of caffeine and minerals, especially K, P, Mg, Ca, Mn and Cu. The total melanoidin and 5-HMF concentration was also higher in the cold-brewed samples, but the concentrations were considered safe for human health.

The temperature during the cold brewing process affected the chemical composition of the cascara beverage. Brewing at 20 °C (room temperature) has proven to be highly effective. This method results in a beverage with a high caffeine concentration, which is the main reason for its consumption. Additionally, brewing at this temperature does not require a cooling medium, ensuring safe energy consumption. Moreover, cold-brewed cascara can be an interesting addition to the non-alcoholic beverage market. Its unique flavour profile, high caffeine and copper content make it a compelling alternative to traditional caffeinated drinks. This innovative product could appeal to consumers seeking new and exciting beverage options. Future research could explore the long-term benefits of regular consumption of cold-brew cascara, as well as its potential applications in functional beverages.

## Appendix

**Table S1:** The effect of brewing methods on the concentration of melanoidins in cascara samples

	Hot-brewed	Cold-brewed at *t*/°C				
Compound		20	15	10	5	p
	*γ*(compound)/(mg/L)					
5-HMF	(0.91±0.03)^b^	(0.95±0.01)^a^	(0.93±0.04)^ab^	(0.96±0.04)^a^	(0.97±0.02)^a^	NS
Furfural	(0.18±0.01)^ab^	(0.16±0.04)^b^	(0.19±0.01)^a^	(0.20±0.01)^a^	(0.19±0.01)^a^	NS
TMC/10^3^	(3.4±0.3)^b^	(4.9±0.4)^a^	(5.1±0.3)^a^	(5.1±0.4)^a^	(5.12±0.08)^a^	NS

Mean value±standard deviation (*N*=3). 5-HMF=5-hydroxymethylfurfural, TMC=total melanoidin content expressed as caramel. different lower-case letters in superscript in the same row indicate statistical differences using Duncan´s multiple pairwise test (p<0.05). NS=the effect of cold brewing temperature on variables was not significant
